# Effects of Exercise Training on Physical Activity in Older People: a Randomized Controlled Trial

**DOI:** 10.2188/jea.13.120

**Published:** 2007-11-30

**Authors:** Kazuki Fujita, Ryoichi Nagatomi, Atsushi Hozawa, Takayoshi Ohkubo, Koya Sato, Yukiko Anzai, Catherine Sauvaget, Yoko Watanabe, Akira Tamagawa, Ichiro Tsuji

**Affiliations:** 1 Division of Epidemiology, Department of Public Health and Forensic Medicine, Tohoku University Graduate School of Medicine.; 2 Department of Medicine and Science in Sports and Exercise, Tohoku University Graduate School of Medicine.; 3 Division of International Health, Department of Public Health and Forensic Medicine, Tohoku University Graduate School of Medicine.; 4 Research Unit of Public Health Nursing, Miyagi University School of Nursing.; 5 Department of Epidemiology, Radiation Effects Research Foundation.; 6 Department of Behavioral Medicine, Tohoku University Graduate School of Medicine

**Keywords:** older adults, total daily energy expenditure, METs

## Abstract

Background: Recent randomized controlled trials indicated that exercise training for elderly significantly increased their physical fitness. However, very few studies have examined changes in physical activity after exercise training. The purpose of this study was to investigate whether six-month exercise training for older adults can increase and maintain their physical activity in daily life.

Methods: Sixty-two men and women aged 60 to 81 years (mean age 67.1 years), living in communities, were randomly allocated into an exercise group (n=32) or a control group (n=33). The intervention started in April 1998 and lasted for 25 weeks. The exercise regimen consisted of endurance training and resistance exercises in a two-hour class conducted at least twice a week. The subjects completed a physical activity diary at each pre-intervention (March 1998), post-intervention (September 1998) and follow-up (April 1999) measurement of physical activity. Physical activity, expressed as total daily energy expenditure, was calculated by multiplying the amount of time spent in each activity and the corresponding METs.

Results: Total daily energy expenditure significantly increased from 40.8 kcal/kg/day to 43.5 kcal/kg/day in the exercise group (p=0.03), but did not change in the control group. At the follow-up measurement, the mean total daily energy expenditure in the exercise group remained significantly higher, by 1.7 kcal/kg/day, than that at the pre-intervention (p=0.05).

Conclusions: This randomized controlled trial indicated that exercise training for elderly was effective in increasing physical activity in daily life.

Recent randomized controlled trials have indicated that exercise training for older adults significantly increased their aerobic capacity^1^^,^^2^ muscle strength,^3^^-^^7^ and so forth. For instance, Blumenthal^1^ and Posner^2^ reported that four-month aerobic exercise training for men and women aged 60 years and older significantly increased peak oxygen consumption by 8.5-15%. Fiatarone reported that 10-week resistance training for very elderly adults (mean age 87.1 years) approximately doubled lower limb muscle strength. ^3^ We also conducted six-month exercise training for older Japanese adults, and the maximum oxygen consumption in the exercise group increased significantly, by 6.4%.^8^

In order to maximize health benefits of exercise training, it is necessary to increase and maintain physical activity in daily life. However, very few studies have examined changes in physical activity after exercise training in older adults. In the trial by Fiatarone, older adults significantly increased their daily physical activities after the training.^3^ However the authors did not investigate whether this increase persisted after the training period. Two other randomized controlled trials, Project Active^9^^,^^10^ and the walking trial^11^, agreed that exercise group subjects significantly increased their physical activity and that this increase was maintained even after 24 months. However, these trials included younger subjects, and the effect of exercise training on daily physical activity in elderly subjects has not been fully ascertained. The objective of this study is to investigate how daily physical activity changes after exercise training in older adults. We measured total daily energy expenditure by the physical activity diary method, and compared the values between the exercise and control groups before and after a six-month exercise intervention.

## METHODS

The Sendai Silver Center is a health and welfare facility for older adults, run by the Sendai City Health and Welfare Foundation. This randomized controlled trial was designed to evaluate the effectiveness of six-month training for older adults living in the community. The details of this trial have been reported elsewhere.^8^^,^^12^ In short, we recruited volunteers in February 1998. Inclusion criteria were men and women aged 60 years or older, living in Sendai City. Exclusion criteria were as follows: (1) moderate to severe motor impairment or neurological deficit; (2) history of coronary heart disease within six months before the study; (3) systolic blood pressure ≧ 160mmHg or diastolic blood pressure ≧ 100mmHg; (4) joint pain or arthritis; (5) mental or other conditions possibly interfering with participation; (6) other chronic disease possibly interfering with participation; (7) history of fracture of a lower extremity or injurious falls within six months before the study; (8) taking antihypertensive agents; and (9) presence of cardiovascular abnormality revealed by exercise testing.

Out of 209 possible participants, we excluded 144 subjects according to the above criteria. After the pre-intervention measurement at the end of March 1998, we randomly allocated 65 subjects (30 men and 35 women; mean age = 67.1, range 60 to 81 years) into either an exercise group or a control group. The intervention lasted for 25 weeks, starting the first week of April 1998. We conducted the post-intervention measurement on both groups at the end of September 1998. Furthermore, we repeated this measurement at the beginning of April 1999 (follow-up measurement).

Written informed consent was provided by all the subjects. The protocol and all ethical aspects were approved by the Executive Board of the Sendai Health and Welfare Foundation.

The subjects of the exercise group received three 2-hour classes a week at the Sendai Silver Center. They were asked to attend the classes at least twice a week. The main session consisted of endurance training using a bicycle ergometer and resistance exercises using flex-bands. The details of this exercise training have been described elsewhere.^8^

The subjects cycled at 50-60 rpm on a bicycle ergometer for 10-25 min under an individually prescribed workload. The exercise intensity was set according to the American College of Sports Medicine guidelines for exercise prescription.^13^ We determined each subject’s target heart rate using the heart rate reserve method, based on the subject’s age-adjusted maximum heart rate (220 minus age [year]), and his or her resting heart rate.

Heart rate reserve = maximum heart rate – resting heart rate,

Target heart rate = heart rate reserve × 50∼60% + resting heart rate.

The exercise group subjects underwent a regimen of progressive resistance training consisting of sit-ups for the trunk flexors and five exercises using the flex-band (Thera-Band^®^ Resistive Exerciser, Hygenic Corp., Akron, Ohio); (1) side-raise for supraspinatus and deltoid muscles, (2) elbow flexion for biceps muscles, (3) knee extension for quadriceps muscles, (4) hip abduction, and (5) hip adduction. In the sit-up, the number of repetitions was progressively increased in the following stages: 10 repetitions at 1-5 weeks; 12 repetitions at 6-7 weeks; 15 repetitions at 8-19 weeks; 20 repetitions at 20-25 weeks. In the five types of exercises using the flex-band, we prescribed exercise at a load with which each subject could repeat the exercise 20 repetitions. The load of each exercise was progressively increased in the 6th, 11th, 16th, 20th and 23rd weeks, respectively, with 1-week recovery periods in the 15th, 19th, and 22nd weeks.

The control group subjects received two 2-hour classes a month at the Sendai Silver Center. They were asked to attend classes at least once a month. The classes consisted of a 1-hour lecture, the topic of which was not related to physical activity, and a 1-hour seated recreational activity such as playing games. Otherwise, they were asked to continue their usual way of life. For ethical reasons, we also provided the control subjects with a six-month exercise training regimen after the post-intervention measurement. However, we did not assess their physical activity at the follow-up measurement.

We gave the subjects of both groups a physical activity diary and asked them to write their activities every 15 min for the waking interval. The physical activity diary consisted of a major activity section and a strenuous activity section. In the major activity section, the subjects were asked to report the activities in which they spent most of the time during each interval. In the strenuous activity section, they were asked to report the most strenuous activity they did irrespective of its duration. The subjects completed the physical activity diary for three consecutive weekdays at each pre-intervention (March 1998), post-intervention (September 1998) and follow-up (April 1999) measurement. In order to improve the accuracy of physical activity information, one of the authors (K.F.) interviewed the subjects to review their responses after they had reported the diary contents.

Total daily energy expenditure (TDEE), expressed as kcal/kg/day, was used as the main outcome measure. We allocated METs (metabolic equivalents) to each physical activity, using the compendium of Ainsworth.^14^ The TDEE was calculated by multiplying the amount of time spent in each activity and the corresponding METs. Furthermore, all physical activities were classified into either light (METs<3.0), moderate (3.0≦METs≦5.0), or strenuous (5.0<METs) activity, based on the classification of Blair.^15^ The validity and reproducibility of the TDEE in this study have been fully ascertained.^16^

All the subjects in both groups completed the 6-month intervention. One woman in the exercise group failed to attend the post-intervention measurement because she had to look after her daughter who was acutely hospitalized a few days before the measurement. Two women in the control group did not complete the physical activity diary at the pre-intervention measurement. Consequently, we herein report the results of 62 individuals (31 in the exercise and 31 in the control group). The subjects ranged from 60 to 81 years in age (mean 67.1 years), and 14 were 70 years or older.

To assess the maintenance of physical activity, we followed the exercise group subjects until April 1999. One man who did not attend the follow-up measurement was excluded from the analysis. Thus, we report the results of 30 individuals in the exercise group.

The sample size of 62 subjects was estimated to provide a statistical power of 80% with p=0.05 to detect a 2.5 kcal/kg/day between-group difference in the TDEE from the pre-intervention to the post-intervention measurement.

All the analyses were performed on an intention-to-treat basis. The main outcome measure was the net TDEE change in the exercise group, defined as the mean between-group difference in TDEE, calculated as (post-intervention TDEE − pre-intervention TDEE) in the exercise group minus (post-intervention TDEE − pre-intervention TDEE) in the control group. Similarly, we compared the mean between-group differences for each intensity category of daily energy expenditure: light, moderate, and strenuous. We examined statistical differences with Student’s t-test or the paired t-test, as appropriate.

All statistical analyses were conducted with SAS^®^ software.^17^ Mean values are presented along with standard errors (SE). P<0.05 was regarded as statistically significant.

## RESULTS

Table 1 shows the means of the pre-intervention measurement TDEE in the subjects. The mean TDEE did not differ significantly between the exercise and control groups. Taking the exercise and control groups together, the mean TDEE was significantly higher in women (42.8 ± 3.1 kcal/kg/day) than in men (40.2 ± 3.5 kcal/kg/day). The mean TDEE was significantly higher in those aged 60-69 (42.1 ± 3.3 kcal/kg/day) than in those 70 or older (39.4 ± 3.5 kcal/kg/day).

**Table 1.** The means and standard deviations of total daily energy expenditure (TDEE) in the exercise and the control groups at the pre-intervention.

		Exercise group		Control group		p-value^a^
	
		n	TDEE (kcal/kg/day)	n	TDEE (kcal/kg/day)	
All subjects		31	40.8 (3.9)	31	42.2 (3.0)	0.11
Subgroup						
Sex	Men	15	39.2 (4.1)	15	41.1 (2.5)	0.14
	Women	16	42.3 (3.2)	16	43.3 (3.0)	0.38
Age	60 – 69	23	41.2 (3.7)	25	43.0 (2.7)	0.07
	70 –	8	39.6 (4.5)	6	39.2 (1.7)	0.82

^a^ : Student’s t-test.

Standard deviations in parentheses.

Table 2 shows the means of TDEE for each group before and after intervention. TDEE significantly increased from 40.8 kcal/kg/day to 43.5 kcal/kg/day in the exercise group, but did not change in the control group. The net change in TDEE in the exercise group was 2.5 kcal/kg/day (p=0.03), which corresponds to an increase of 6.4% (p=0.03). The significant association between the allocation group and the degree of increased TDEE remained significant after we adjusted for baseline TDEE level of the subjects (p=0.048).

**Table 2.** Comparison of mean of total daily energy expenditure.

Group	n	Before	After	Change		Net change^a^ (95 % CI)	
			
		kcal/kg/day	kcal/kg/day	kcal/kg/day	%	kcal/kg/day	%
				
Exercise	31	40.8 (0.7)	43.5 (1.1)	2.7 (1.0)**	6.9 (2.5)**	2.5 (0.3, 4.7) ^†^	6.4 (0.8, 12.0) ^†^
Control	31	42.2 (0.5)	42.4 (0.7)	0.2 (0.6)	0.5 (1.3)		

^a^ : The difference between the change in the exercise group and the change in the control group.

** p<0.01 vs pre-intervention

^†^ p=0.03

CI : confidence interval

Standard errors in parentheses.

Table 3 shows the means of TDEE according to sex and age groups. The net change in TDEE in men and women was 2.3 ± 1.9 kcal/kg/day and 2.7 ± 1.3 kcal/kg/day, but the difference reached statistical significance in only women (p=0.048). The net change in TDEE was higher in those aged 70 years or older (4.6 ± 2.8 kcal/kg/day) than in those aged 60-69 (1.8 ± 1.1 kcal/kg/day), but the difference was not statistically significant.

**Table 3.** Comparison of mean of total daily energy expenditure according to sex and age group.

	Group	n	Before	After	Change	Net change ^a^
			
			kcal/kg/day	kcal/kg/day	kcal/kg/day	kcal/kg/day
				
*Sex*						
Men	Exercise	15	39.2 (1.1)	41.9 (2.0)	2.7 (1.7)	2.3 (-1.5, 6.1)
	Control	15	41.1 (0.7)	41.5 (1.2)	0.4 (0.9)	

Women	Exercise	16	42.3 (0.8)	45.0 (1.1)	2.7 (1.1)*	2.7 (0.1, 5.3)*
	Control	16	43.3 (0.8)	43.3 (0.7)	0.0 (0.7)	

*Age*						
60-69	Exercise	23	41.2 (0.8)	43.0 (1.2)	1.8 (0.9)	1.8 (-0.4, 4.0)
	Control	25	43.0 (0.5)	43.0 (0.8)	0.0 (0.6)	

70-	Exercise	8	39.6 (1.6)	45.0 (2.8)	5.5 (2.6)	4.6 (-1.0, 10.2)
	Control	6	39.2 (0.7)	40.0 (0.8)	0.9 (1.2)	

^a^ : The difference between the change in the exercise group and the change in the control group.

* : p<0.05

Table 4 shows the means of the sum of physical activity time classified by three intensity levels. The light activity time in the exercise group significantly decreased (-46.7 minutes, p=0.049) while both moderate and strenuous activity time tended to increase (40.0 minutes and 6.7 minutes, respectively), as compared with pre-intervention levels. Most of the increased physical activity was attributable to an increase in moderate activity.

**Table 4.** Comparison of mean of the sum of physical activity time according to the level of intensity.

	Group	n	Before	After	Change	Net change ^a^
			
			minutes	minutes	minutes	minutes
				
Light	Exercise	31	1302.7 (10.0)	1256.0 (18.6)	-46.7 (20.0)*	-47.2 (-94.1, -0.4)*
	Control	31	1277.8 (8.9)	1278.3 (11.5)	0.5 (12.2)	

Moderate	Exercise	31	127.0 (9.6)	167.0 (18.7)	40.0 (20.1)	39.4 (-7.6, 86.3)
	Control	31	151.7 (8.2)	152.3 (10.5)	0.6 (12.2)	

Strenuous	Exercise	31	10.3 (2.7)	17.0 (5.4)	6.7 (6.0)	7.9 (-5.2, 21.0)
	Control	31	10.6 (2.5)	9.4 (3.0)	-1.2 (2.6)	

a : The difference between the change in the exercise group and the change in the control group.

* : p<0.05

We further compared the frequency of the subjects who increased their physical activity by 2.0 kcal/kg/day or more, which met the recommendation by U.S. Surgeon General.^18^ The frequency was significantly higher in the exercise group, that is 69% in the exercise group and 31% in the control group (p=0.01).

We observed whether increased physical activity in the exercise group was maintained after completion of the training session (Figure). Although the TDEE at the follow-up measurement had decreased by 1.2 kcal/kg/day as compared to the post-intervention TDEE, it remained significantly higher by 1.7 kcal/kg/day than the pre-intervention TDEE (p=0.05). The daily energy expenditure of moderate activity at the follow-up measurement also remained significantly higher by 2.5 kcal/kg/day than the pre-intervention daily energy expenditure (p=0.02), although the strenuous activity had returned to the pre-intervention level by the time of final follow-up.

**Figure.**
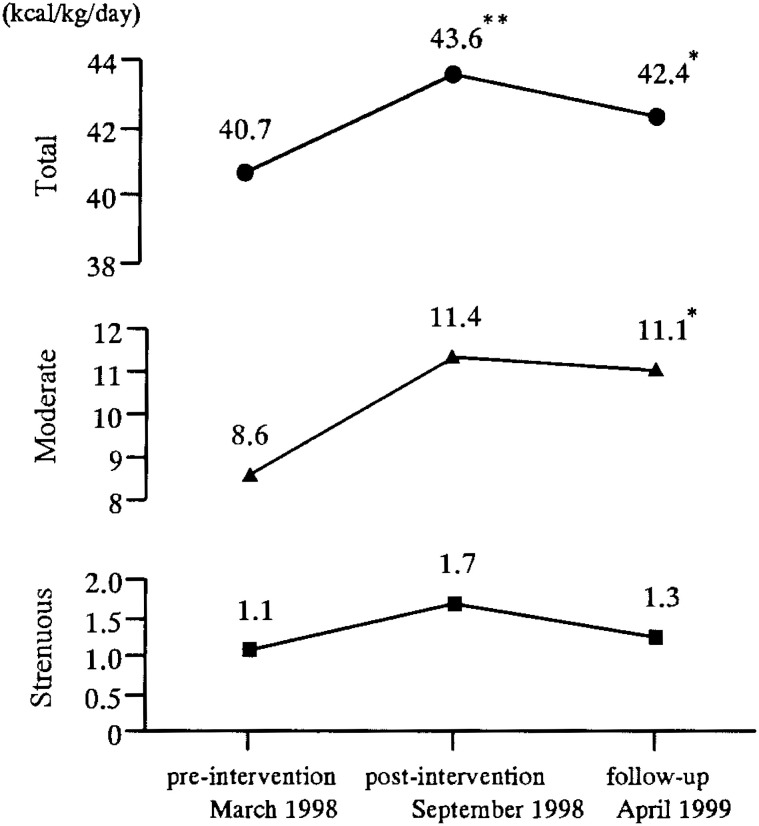
Maintenance of physical activity from the pre-intervention to follow-up measurement in the exercise group. * : p<0.05 vs pre-intervention ** : p<0.01 vs pre-intervention

## DISCUSSION

The present randomized controlled trial was designed to evaluate the effectiveness of 6-month exercise training for older adults. Although we did not instruct our subjects to increase physical activity in their daily lives, the exercise group subjects significantly increased their spontaneous physical activity and this increase was maintained as long as 6 months after the completion of training. Another important point in the present study is that the degree of the increase in physical activity tended to be larger in those 70 years and over than in those 60-69 years. This finding underscores the fact that it is never too late to start exercising, no matter how old you are.

Only a few randomized controlled trials have investigated changes in spontaneous physical activity after exercise training among middle-aged or older subjects living in the community. Kriska^11^ conducted a 2-year walking intervention program for postmenopausal women. The walking group had significantly increased their physical activity, by 45 kcal per day, at the end of the intervention program. Dunn^9^ compared the change in physical activity between lifestyle intervention and structured exercise intervention (Project Active). Our exercise group was similar to the structured exercise group in Project Active. The gain in physical activity after a 6-month exercise intervention was larger in our trial (2.5 kcal/kg/day) than in Project Active (1.3 kcal/kg/day). The TDEE increase of 2.5 kcal/kg/day in the exercise group, which is equivalent to one hour usual walking or 30-40 minutes brisk walking, meets the recommendation of U.S. Surgeon General that an energy expenditure increase of 2 kcal/kg/day would be sufficient to change sedentary adults to moderately active.^18^

If increased physical activity after exercise training was not maintained for a long period, exercise training would have little impact on health. Six months after completion of exercise training, we found that TDEE and moderate activity remained significantly higher than the pre-intervention values, although strenuous activity had returned to the pre-intervention level. Thus, an appropriate exercise program should aim at increasing and maintaining the amount of moderate activity in daily life, which meet the recommendation made by the Centers for Disease Control and Prevention and the American College of Sports Medicine^19^ and the National Institute Health^20^, promoting the incorporation of moderate activity into daily life.

This study has several limitations. First, we selected the subjects according to several exclusion criteria which were related to conditions interfering with participation and safety in the exercise training. Therefore, our subjects were healthier and more active than the general population at their age. We must consider whether the present results are generalizable to frail and sedentary older adults. Second, there was no data at 6 months after the completion of the training in the control group. Therefore, the present study did not actually address the question as to whether an exercise training results in persistent or long-term increase in physical activity. Third, we estimated physical activity on the basis of self-reported physical activity diary of the subjects. Therefore, the following is possible: the subjects in the exercise group were more likely to overreport their physical activity; the subjects in the exercise group were more likely to recall their physical activity in detail.

In conclusion, the present randomized trial demonstrated that exercise training for older adults effectively increased their physical activity in daily life, which was maintained for 6 months after the completion of the training session. Future study would be needed to determine whether the increased physical activity would last for a longer period. If the increased physical activity is maintained for a long time after completion of the training session, the elderly will experience enormous benefits in terms of maintaining their health and physical functioning, thereby extending the span of active and healthy life.

## APPENDIX

List of SSCT investigators and committees

Principal investigator: Ichiro Tsuji ^1^.

Advisory Committee: Shigeru Hisamichi ^1^, Hiroaki Ohmori ^2^, Hideo Hashimoto ^3^, Noboru Kokubo ^3^, Shinichi Shoji ^3^, Kei Kudo ^4^.

Project Office: Tomohiko Konno ^3^, Noriko Irie ^3^, Masahiro Saito ^3^.

Randomized Allocation Committee: Yoshikazu Nishino ^1^, Keiko Suzuki ^1^.

Eligibility Committee: Catherine Sauvaget ^5^, Seiki Kanemura ^1^, Aya Kuwahara ^1^, Chikako Nakagawa ^1^, Peng Hua Qiang ^1^.

Measurement Committee: Akira Tamagawa ^6^, Takayosi Ohkubo ^1^, Akira Sato ^2^, Keiko Ogawa ^2^, Yoshitaka Tsubono ^1^, Tasuku Sato ^7^, Masafumi Ohsako ^8^, Tetsuro Suzuki ^8^, Yoko Watanabe ^1^, Yuko Yoshida ^9^, Mitsuharu Okutsu ^2^, Zhang Xiu Ming ^2^, Osamu Kanemi ^2^, Koya Sato ^2^, Hirotoshi Sasaki ^2^, Akira Sato ^2^, Yutaka Imai ^10^.

Training Committee: Ryoichi Nagatomi ^2^, Kazuki Fujita ^1^, Mie Harada ^3^, Eiko Kitame ^3^, Reiko Konishi ^3^, Midori Neda ^3^, Megumi Watanabe ^3^.

Safety and Monitoring Committee: Atsushi Hozawa ^1^, Yukiko Anzai ^4^, Setsuko Goto ^3^, Junko Tanaka ^3^.

^1^ Division of Epidemiology, Department of Public Health and Forensic Medicine, Tohoku University Graduate School of Medicine.
^2^ Division of Medicine & Science in Sports & Exercise, Department of Behavioral Science, Tohoku University Graduate School of Medicine.
^3^ Sendai Silver Center, Sendai City Health and Welfare Foundation.
^4^ Research Unit of Public Health Nursing, Miyagi University School of Nursing.
^5^ Department of Epidemiology, Radiation Effects Research Foundation.
^6^ Division of Behavioral Medicine, Department of Behavioral Science, Tohoku University Graduate School of Medicine.
^7^ Department of Sports Science, Faculty of Physical Education, Sendai College.
^8^ Department of Sports and Health Science, Course of Liberal Arts, Toyo University.
^9^ Department of Community Health, Tokyo Metropolitan Institute of Gerontology.
^10^ Department of Clinical Pharmacology and Therapeutics, Tohoku University Graduate School of Medicine and Pharmaceutical Science.
